# Healthy Leaders: Core Self-Evaluations Affect Leaders’ Health Behavior Through Reduced Exhaustion

**DOI:** 10.3389/fpsyg.2019.00998

**Published:** 2019-07-10

**Authors:** Christina Köppe, Astrid Schütz

**Affiliations:** Competence Centre for Personnel Psychology, University of Bamberg, Bamberg, Germany

**Keywords:** leaders, core self-evaluations, health behavior, exhaustion, organizational health climate

## Abstract

Leaders’ self-directed health behavior (i.e., SelfCare behavior) plays an important role in the health and well-being of both leaders and employees but has been neglected in research so far. This study was aimed at investigating the antecedents of SelfCare behavior in terms of the personal characteristics of the leaders. In a sample of 150 (98 male, 52 female) German leaders from a wide range of organizations, we examined the direct and indirect effects of core self-evaluations (i.e., CSEs) on leaders’ SelfCare behavior. We predicted that CSEs would be positively related to SelfCare behavior with reduced exhaustion as a mediator, and organizational health climate (i.e., OHC) as a moderator of this relationship. Results showed that CSEs were positively related to SelfCare behavior and that the reduced exhaustion mediated this relationship. There was no evidence that OHC moderated the positive relationship between CSEs and SelfCare behavior. Theoretical and practical implications of the study are discussed.

## Introduction

Leadership positions are characterized by high job demands. For example, leaders often have to deal with a large workload, time pressure, or role conflicts (Ohm and Strohm, 2001; Zimber et al., 2015). According to the job demands-resources model (Demerouti et al., 2001; Schaufeli and Taris, 2014), high job demands predict poor health. Indeed, leaders who suffer from impaired health are less able to care for their employees. In this vein, Harms et al. (2017) showed that leaders engage less in high-quality leadership, such as transformational leadership (Burns, 1978; Bass, 1985), when they feel exhausted and stressed. This potential lack of transformational leadership is consequential for their work environment because the leadership style has been found to have a positive influence on employees’ work-related well-being (for a review, see Skakon et al., 2010).

Given that leaders’, as well as employees’, health is constantly at risk, researchers have called for interventions that can empower leaders when dealing with the challenges of their role and their own health (Zwingmann et al., 2016; Harms et al., 2017). The health-oriented leadership (HoL) approach by Franke et al. (2014) introduces a concept that focuses on leaders’ self-directed health-promoting behavior (i.e., SelfCare). SelfCare is aimed at protecting or promoting one’s own health by “dealing appropriately with job demands and fostering health-promoting working conditions” (p. 142) and should thus be the focus of health-promoting interventions for leaders. SelfCare is understood as the extent to which leaders are aware of their own health, value its importance, and behave accordingly (Franke et al., 2014). Although all three components are supposed to be relevant aspects of the SelfCare concept, the behavioral component seems to be particularly consequential because it reflects the extent to which leaders actually engage in health-relevant actions (e.g., seeking social support, balancing work, and leisure time).

To date, research on the SelfCare behavior of leaders has been almost nonexistent. As an exception, Franke et al. (2015) illustrated that leaders’ SelfCare is associated with better health, less irritation, and lower levels of work-family conflicts, underlining its effectiveness. But studies focusing on the antecedents of the SelfCare behavior of leaders have been rare, even though investigating the factors that may impact leaders’ health behavior may help to promote such behavior. In searching for antecedents, Booth-Kewley and Vickers (1994) found that “personality is a reliable predictor of health behavior” (p. 281). Self-efficacy in particular has received a lot of attention in research and was found to play a pivotal role in health behavior (e.g., Luszczynska and Schwarzer, 2003). Closely intertwined with self-efficacy are locus of control and self-esteem. Studies have found that both variables are closely related to positive health behaviors (e.g., Booth-Kewley and Vickers, 1994; Christian et al., 2009). Moreover, several studies have focused on the Big Five as predictors of health behavior (e.g., Booth-Kewley and Vickers, 1994; Bogg and Roberts, 2004). Neuroticism has been intensively studied and was found to be associated with risky health behaviors such as smoking or alcohol abuse (e.g., Vollrath and Torgersen, 2002). Self-efficacy, locus of control, self-esteem, and neuroticism represent the core facets of the higher order factor CSEs. Although each core trait can be linked to health behavior, CSEs as a whole seems to have special relevance with respect to leadership behavior (Resick et al., 2009). Still it is not yet known how that higher order factor is related to leaders’ self-directed health behavior.

Personality is a relevant predictor of behavior, but its effects cannot be observed in every situation. Interactionist approaches (see Mischel and Shoda, 1995) and the more recent elaboration of trait activation theory (TAT; Tett and Burnett, 2003) suggest that personality traits have to be activated by situational cues in order to trigger situation-specific behavior. According to TAT, situational cues occur on three different levels: task, social, and organizational. In the workplace, organizational cues are important because they indicate which behaviors are accepted by the organization and which are not. OHC is a prominent example of a powerful organizational cue and should be relevant to individuals’ health behavior in organizations.

**FIGURE 1:**
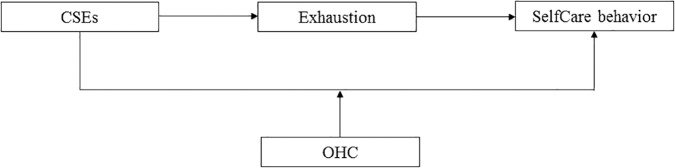
Theoretical model of the relationship between CSEs and SelfCare behavior. CSEs, core self-evaluations; OHC, organizational health climate.

In this study, we investigated the direct as well as indirect effects of CSEs on leaders’ SelfCare behavior. Furthermore, we examined the moderating role of OHC in the relationship between CSEs and SelfCare behavior. We tested this theoretical model (see Figure 1) in a sample of leaders. In doing so, we expected this study to contribute to the existing literature in two ways: First, concentrating on the personal antecedents (i.e., CSEs) of leaders’ SelfCare behavior is important for preventing illness and promoting health behavior in leaders, which in turn can affect the health and well-being of their employees. Second, because we also considered situational factors in the personality-health behavior link, this study can offer a starting point for the development of organizational measures.

### Effects of CSEs on SelfCare Behavior

Core self-evaluations represent a broad higher order personality trait that is based on “the conceptual and empirical overlap of four dispositional traits: neuroticism, self-esteem, self-efficacy, and internal locus of control” (Judge and Bono, 2001; Judge et al., 2003; Stumpp et al., 2010, p. 675). CSEs reflect “a basic, fundamental appraisal of one’s worthiness, effectiveness, and capability as a person” (Judge et al., 2003, p. 304). Thus, people with high CSEs see themselves in a more positive light than those with low CSEs. These fundamental appraisals about oneself influence how people behave in specific situations and how motivated they are to do so consistently (see Hu et al., 2012).

In terms of health behaviors, the four core traits play a crucial role in how people care for themselves. The relation between each of those specific traits and health behavior has been studied: Self-esteem and self-efficacy both represent self-evaluative tendencies (Schütz, 2001; Lanaj et al., 2012). Whereas self-esteem is defined as the overall evaluation of one’s own worth, self-efficacy refers to one’s perceived ability to cope with difficulties and to perform well in challenging situations (Bandura, 1977; Judge and Bono, 2001). People with high self-esteem report being happier, tend to experience greater control, and have better coping abilities (Baumeister et al., 2003). Besides, people in general tend to protect and enhance their self-esteem in order to feel good about themselves (Baumeister, 2010; Schütz and Baumeister, 2017). In line with this, Lanaj et al. (2012) found a positive association between self-esteem and promotion focus, thus illustrating its motivational background (see also Schütz and DePaulo, 1996). From this perspective, people with positive self-views tend to value themselves and engage in health-oriented behaviors, as they usually work toward positive outcomes such as happiness, control, or health. There is a lot of research in which health-relevant behaviors (e.g., smoking or alcohol and drug abuse) have been studied in relation to self-esteem (e.g., McGee and Williams, 2000). Although the findings are somewhat mixed, several studies have reported a positive relationship between congruent positive self-evaluations and a general tendency to engage in positive health behaviors (e.g., Schröder-Abé et al., 2007).

Self-efficacy is embedded in various theories on health behavior such as the transtheoretical model (Prochaska and DiClemente, 1983) or the health action process approach (Schwarzer, 1992). Both theories suggest that self-efficacy is a central factor in promoting health behavior. If people believe they are capable of performing a certain health behavior, they are more likely to start it and keep it up (Schwarzer and Fuchs, 1995). In this vein, in sample of 418 young women, Luszczynska and Schwarzer (2003) found that self-efficacy rather than risk perception was the best predictor of the intention to perform preventive behaviors (i.e., breast self-examination).

Locus of control has also been applied to predict health behaviors. Locus of control refers to the degree to which a person believes that the consequences of his or her behavior are due to internal (e.g., ability or other personal characteristics) or external (e.g., luck or fate) factors (Rotter, 1966). Besides, a study showed that locus of control can be domain-specific and applied to health beliefs (Wallston et al., 1978). The idea behind this refers to the general assumption that “individuals who believe that they have control over their health (internal health locus of control) will be more likely to engage in health-enhancing behaviors” (Norman et al., 1998, p. 172, authors’ supplement). In line with this, Norman et al. (1998) showed that people who have a strong internal health locus of control performed a higher number of health behaviors (e.g., exercising three times a week, eating fruits at least 6 days a week). Moreover, Christian et al. (2009) found that locus of control was positively related to safety-related behaviors (in terms of safety compliance and safety participation) in a workplace setting. In their study, the authors also included other personality variables such as neuroticism. Neuroticism, or emotional instability, means that individuals experience more negative than positive emotions and that they are prone to mood swings (Ostendorf and Angleitner, 2004). People high in neuroticism are more likely to engage in harmful health practices and less likely to show health-promoting behavior than others. For example, Booth-Kewley and Vickers (1994) showed that people with high scores in neuroticism reported less wellness-oriented behavior (i.e., exercising, eating healthy food), less accident-control behavior (i.e., fixing broken things, having a first aid kit), and more traffic-related risk-taking (i.e., speeding, not obeying traffic rules) than others. Along these lines, Christian et al. (2009) found that locus of control was negatively related and neuroticism was positively related to accidents and injuries.

Although each core trait can be linked to health-oriented behaviors, a higher order factor comprised of CSEs has not yet been studied and especially not with a sample of leaders. Ferris et al. (2011) argued that the reason why CSEs affect a broad range of outcomes (i.e., job satisfaction, work commitment, or stress) can be attributed to an overall approach/avoidance framework, thus suggesting that, as opposed to low-CSE individuals, people with high levels of CSEs have an overall approach tendency (for a similar argument see Tice, 1991; Higgins et al., 1994). It can be assumed that high-CSE leaders tend to view themselves as worthy, competent, capable, and in control of their own health, all of which motivate them to actively engage in self-directed health behaviors. In line with this reasoning, meta-analytic results revealed that CSEs are significantly related to intrinsically motivated behavior (Chang et al., 2012). In addition, Kammeyer-Mueller et al. (2009) found that high-CSE individuals engaged more in problem-solving coping and less in avoidance coping. It can be argued that problem-solving coping bears a resemblance to SelfCare behavior. Coping as such refers to a person’s intention to engage in certain actions that are aimed at reducing threat or harm in a given situation (Lazarus and Folkman, 1984). Problem-focused coping in particular refers to a kind of coping that is targeted toward changing the stressful situation or the source of stress. Thus, people who use problem-solving coping may, for example, try to alter their working conditions, strategies, or time schedules. Likewise, SelfCare behavior means promoting health by enhancing health-promoting working conditions.

Taken together, we propose the following:

*Hypothesis 1: CSEs will be positively related to SelfCare behavior*.

### OHC as a Moderator of the Relationship Between CSEs and SelfCare Behavior

There is a long history of research that has focused on organizational climate (for a review see Schneider et al., 2017). There are various definitions of organizational climate that can be integrated by stating, “it is a summary perception derived from a body of interconnected experiences with organizational policies, practices and procedures” (Schneider et al., 2017, p. 468). Organizational climate is a very broad construct that lacks specificity and thus cannot easily be used to predict certain specific outcomes (Schneider and Snyder, 1975). Thus, Schneider and Snyder (1975) called for the study of specific climates, such as a climate for safety. In our study, we focused on a *perceived* organizational climate. We emphasize the term perceived as we did not include objective measures in our study, and we analyzed climate on an individual level. Accordingly, OHC refers to the employee’s perception that the policies, practices, and procedures applied by the organization are important with respect to health within this organization (Gurt et al., 2011).

Organizational climate is supposed to affect individuals’ behavior in organizations (Glick, 1985). Building on TAT (Tett and Burnett, 2003), we suggest a moderating effect of OHC in the relationship between CSEs and SelfCare behavior. TAT was originally developed to explain how traits are related to work behavior in terms of job performance. TAT represents a person-situation interactionist model that emphasizes that “traits are expressed in work behavior as responses to trait-relevant situational cues” (Tett and Burnett, 2003, p. 503). According to the model, situational cues are moderators that specify when and how a certain trait is expressed. Tett and Burnett (2003) distinguished between three sources of trait-relevant situational cues provided in work settings: task-related, social, and organizational cues. Situational cues on the organizational level are represented in organizational culture and climate (Tett and Burnett, 2003). OHC can be seen as a relevant cue for activating personality traits, which in turn promote certain work-related behaviors (i.e., SelfCare behavior in this case). In the present study, we focused on the organizational level as it seems especially relevant to health behaviors.

However, Tett and Burnett (2003) stressed that situational cues need to be “trait-relevant” (p. 502) to give rise to trait activation. Therefore, an organizational climate for health has to be connected to CSEs in such a way that responses to the relevant cue “indicate a person’s standing on the trait” (Tett and Burnett, 2003, p. 502). First, people with high levels of CSEs – due to an approach tendency and promotion focus (Higgins, 1998; Ferris et al., 2011) – are supposed to be more sensitive to positive stimuli and less sensitive to negative stimuli (Ferris et al., 2011; Chang et al., 2012). Thus, positive situational cues should be more salient for high-CSE individuals than low CSE-individuals. High-CSE individuals should therefore be more motivated to engage in certain behaviors that help them to achieve positive outcomes such as happiness, control, or health (Ferris et al., 2011). For example, a situation in which an organization offers employees free opportunities to improve their health (e.g., in terms of health practices such as stress management trainings, or health policies such as flexible working hours or working from home) is relevant to CSEs because responding to such a cue would suggest that people evaluate themselves as worthy, effective, and capable with respect to health-related issues, whereas ignoring such a cue would not. On the other hand, the OHC cue should be less motivating for low-CSE individuals because they are less confident that they can achieve desirable outcomes (Leary and MacDonald, 2003).

Even if there are theoretical reasons to expect a moderating effect of OHC in the relationship between CSEs and SelfCare behavior, to our knowledge such an effect has not yet been tested. In fact, empirical studies focusing on OHC are rather scarce (for an exception see Gurt et al., 2011). By contrast, a lot of research has been conducted on safety climate, a concept that bears some similarities to health climate as it also concerns employees’ climate perceptions and their effects on physical health (Zohar, 2014). In this context, studies have confirmed the moderating role of a positive safety climate (e.g., Hofmann et al., 2003).

Given the theoretical and empirical background described above, we propose the following:

*Hypothesis 2: OHC will moderate the relationship between CSEs and SelfCare behavior such that the relationship will be stronger under high than under low OHC*.

### Exhaustion as a Mediator of the Relationship Between CSEs and SelfCare Behavior

Core self-evaluations have consistently been found to have positive effects on different health-related outcomes (Chang et al., 2012). A meta-analysis by Alarcon et al. (2009), for example, found that individuals with higher CSEs experience less job burnout. Burnout can be defined as a multidimensional construct that comprises three core dimensions: (emotional) exhaustion, cynicism, and (reduced) personal accomplishment (Maslach et al., 2001). However, in comparison with cynicism and (reduced) personal accomplishment, exhaustion can be seen as the most central dimension of burnout (Maslach et al., 2001); it refers to feelings of being strained and depleted by one’s job (Demerouti et al., 2003). Therefore, we concentrated on exhaustion as a potential mediator in this study.

The positive impact of CSEs on exhaustion can be explained by the way people perceive their environment regarding different job characteristics (differential exposure hypothesis; Kammeyer-Mueller et al., 2009). High-CSE individuals tend to experience their work environment as challenging rather than threatening and should therefore feel less exhausted as compared with people with low levels of CSEs. In support of this assumption, Kammeyer-Mueller et al. (2009) found that people with high CSEs reported fewer stressors than people with low CSEs did. Leaders who feel energetic and fit are in turn supposed to engage in self-directed health behavior more often than leaders who feel depleted and drained. Conservation of resources (CoR) theory offers a theoretical framework to explain why leaders’ exhaustion may impact their SelfCare behavior. According to that theory, “people strive to retain, protect, and build resources” (Hobfoll, 1989, p. 516). The model of conservation of resources further assumes that “individuals are motivated to gain resources. This motivation drives people to invest resources in order to enrich their resource pool” (Hobfoll, 1989, p. 520). Thus, the less exhaustion a person experiences, the more resources he or she should have, and this in turn should lead to increased SelfCare behavior. By contrast, people who lack resources should be more prone to further loss of resources and thus tend to be motivated to protect the resources they have left. Consequently, instead of investing their resources in health behaviors, which by itself cost additional resources, exhausted leaders may take a “defensive posture” (Hobfoll, 2001, p. 356) to protect their resources. To sum up, we propose the following:

*Hypothesis 3: CSEs will affect SelfCare behavior via exhaustion such that higher CSEs will decrease exhaustion while subsequently leading to higher SelfCare behavior*.

## Materials and Methods

### Procedure and Sample

The study was part of a larger research project on the antecedents and effects of health-oriented leadership. Data were collected in Germany from May to September 2016.

The study was advertised through various mailing lists and networks. Specifically, we promoted participation in the study in various ways, for example, through the quarterly newsletter of our Competence Center for Personnel Psychology^1^, the university’s homepage, and its press department. In addition, leaders in the authors’ networks were contacted via email and asked to distribute the web link to the study. To increase their motivation to participate, participants were offered different kinds of incentives: (a) an information sheet on how to lead oneself and one’s employees in a health-supporting manner, (b) a summary of the study results, (c) the chance to win personalized feedback about their ability-based emotional intelligence quotient, as assessed with the Mayer-Salovey-Caruso Emotional Intelligence Test (MSCEIT; Steinmayr et al., 2011). The study was conducted in line with the ethical guidelines by APA. An ethics approval was not required as per our institution’s guidelines and national regulations. Participants provided informed consent to participate by virtue of survey completion.

Using the different recruitment channels resulted in 621 people who clicked on the study link and 306 of those started the study. A total of 181 participants completed the full survey. They were asked to state if they currently hold a leadership position and which management level they belong to. Thirty-one participants had to be excluded due to previously defined exclusion criteria such as working part-time, being self-employed, or not currently holding a leadership position; two participants were excluded because of symmetrical answer patterns. Thus, 150 leaders (98 male, 52 female) of various occupational fields – mostly from manufacturing or merchandising industries – made up the final sample.

Leaders were, on average, 47 (*SD* = 9.12) years old and had been working at their current company for approximately 15.2 (*SD* = 10.38) years, most of them in HR management. Seventeen percent belonged to the lower management, 47% belonged to the middle management, and 35% belonged to the upper management; 1% did not provide such information. On average, they worked 46.9 h (*SD* = 7.12) per week. Seventy-one percent held a university degree (university and university of applied studies), 15% a university entrance qualification, 14% a secondary general school certificate, and 1% an intermediate secondary school certificate.

### Measures

#### Core Self-Evaluations

To measure the broad trait of CSEs, we used the German version of the Core Self-Evaluations Scale by Stumpp et al. (2010), originally developed by Judge et al. (2003). The scale consists of 12 items with six of them reverse coded. Participants stated the degree to which they agreed with each item on a 5-point Likert-type scale (1 = *not at all*, 5 = *completely*). Example items are “Overall, I am satisfied with myself” and “There are times when things look pretty bleak and hopeless to me” (reversed). Cronbach’s alpha was 0.82.

#### SelfCare Behavior

SelfCare behavior (Cronbach’s alpha = 0.65; e.g., “I try to reduce my demands by optimizing my personal work-life balance, e.g., take regular breaks, avoid overtime”) was assessed with four items from the Health-oriented Leadership (HoL) instrument by Franke et al. (2014). All items were answered on a 5-point rating scale ranging from 1 (*not at all true*) to 5 (*completely true*).

#### Exhaustion

Exhaustion was measured with eight items from the Oldenburg Burnout Inventory (Demerouti and Nachreiner, 1998; Demerouti and Bakker, 2008). Sample items are “After my work, I regularly feel worn out and weary” and “After my work, I regularly feel totally fit for my leisure activities” (reversed). Four items were positively worded, and four items were negatively worded. Positively worded items were recoded so that higher scores would reflect higher exhaustion. Items were scored on a 4-point Likert-type scale (1 = *strongly disagree*, 4 = *strongly agree*). Internal consistency was α = 0.85.

#### Organizational Health Climate

Two items from the short version of the Organizational Health and Safety questionnaire were used to measure OHC (Gurt et al., 2010). Items were scored on a 5-point rating scale ranging from 1 (*not at all true*) to 5 (*completely true*). An example item is “Health initiatives in my organization are either insufficient or inadequate” (reversed). Cronbach’s alpha was 0.77.

#### Control Variables

We assessed gender (1 = *male*, 2 = *female*), age, and tenure as control variables due to theoretical reasons. In general, meta-analyses showed that women are more exhausted than men (Purvanova and Muros, 2010). In addition, Brewer and Shapard (2004) found that age and years of experience was negatively related to exhaustion. Where leaders are concerned, studies showed that female (Kromm et al., 2009; Zimber et al., 2015) and middle-aged leaders (from 30 to 50, e.g., Ohm and Strohm, 2001) are more at risk than others with respect to negative health outcomes. Besides, Ohm and Strohm (2001) found that leaders who have held their current position for a long time, reported to be in worse physiological and psychological health than leaders who are at the beginning of their careers.

### Data Analysis

All analyses were done in SPSS (version 23). In order to test our hypotheses, we used the PROCESS macro provided by Hayes (2013). PROCESS can be applied to conduct conditional process analyses. In this study, we tested a conditional process model (see Figure 1), which depicts mediation of the effect of CSEs on SelfCare behavior through exhaustion, with the direct effect moderated by OHC, by specifying the PROCESS model 5. Estimating the effects of interest, 10,000 bootstrap samples were used to calculate bias-corrected bootstrap confidence intervals. In addition, all standard errors were based on the HC3 estimator.

As unstandardized regression coefficients are the default when the PROCESS macro is used, all variables were standardized prior to our analyses. As a consequence, to test the moderating effect of OHC on the relationship between CSEs and SelfCare behavior, the products were not mean-centered before the analysis.

Age, gender, and tenure were also included as control variables in the preliminary correlational analyses.

## Results

### Preliminary Analyses

We estimated the intercorrelations between the study variables, which are displayed in Table 1. Study variables were significantly correlated in the hypothesized direction. CSEs were positively related to SelfCare behavior (*r* = 0.36, *p* < 0.001) and, similar to previous research (e.g., Alarcon et al., 2009), they were negatively related to exhaustion (*r* = −0.62, *p* < 0.001). Exhaustion in turn was significantly negatively related to SelfCare behavior (*r* = −0.36, *p* < 0.001). As expected, OHC was positively associated with CSEs (*r* = 0.33, *p* < 0.001) and SelfCare behavior (*r* = 0.18, *p* = 0.027), respectively.

**Table 1:** Means, standard deviations, and correlations of the study variables.

Variable	*M*	*SD*	1	2	3	4	5	6	7
1. Age^a^	46.84	9.12	–						
2. Gender^b^	1.35	0.48	−0.19^∗^	–					
3. Tenure^c^	15.22	10.38	0.56^∗∗∗^	−0.10	–				
4. CSEs	3.87	0.50	0.08	−0.26^∗∗^	0.05	(0.82)			
5. SelfCare behavior	3.54	0.72	0.01	−0.06	0.03	0.36^∗∗∗^	(0.65)		
6. Exhaustion	2.21	0.55	−0.05	0.22^∗∗^	−0.11	−0.62^∗∗∗^	−0.36^∗∗∗^	(0.85)	
7. OHC	3.53	0.93	−0.01	−0.13	0.10	0.33^∗∗∗^	0.18^∗^	−0.32^∗∗∗^	(0.77)

*N = 150. Pairwise deletion. Cronbach’s alphas are listed on the diagonal. CSEs, core self-evaluations; OHC, organizational health climate. ^a^Age is coded in years. ^b^Gender is coded as 1 = male, 2 = female. ^c^Tenure is coded in years. ^∗^p < 0.05, ^∗∗^p < 0.01, ^∗∗∗^p < 0.001.*

The control variables age and tenure were not significantly correlated with either the mediator or the outcome variable and were thus not included in further analyses.

Because we used self-report questionnaires to collect data, we tested for common method variance (CMV; Podsakoff et al., 2003). We conducted Harman’s single-factor test (see Tehseen et al., 2017) in SPSS to find out “whether one single factor emerges” (Tehseen et al., 2017, p. 155) which makes up for most of the variance in the data. All items of the study constructs were entered into factor analysis (i.e., principal component analysis, no rotation). The results indicate that CMV does not play a pivotal role in this study: In contrast to one single factor, seven distinct factors were extracted which captured 62% of the total variance. Moreover, the first unrotated factor accounted only for 27% of this variance.

### Tests of Hypotheses

**FIGURE 2:**
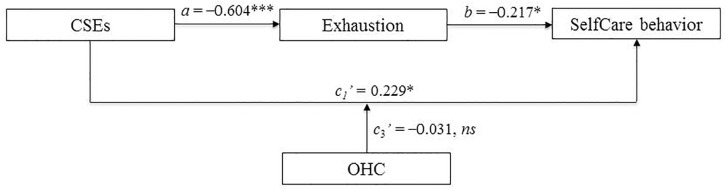
Empirical model of the relationship between CSEs and SelfCare behavior. Standardized regression coefficients are represented in the model. *N* = 149. The effect of gender was controlled for. CSEs, core self-evaluations; OHC, organizational health climate. ^∗^*p* < 0.05, ^∗∗∗^*p* < 0.001.

We will report the results in the order in which the hypotheses were presented (for an overview, see Figure 2). Table 2 shows the study results. Supporting Hypothesis 1, CSEs were positively associated with SelfCare behavior, *c*_1_’ = 0.229, *p* = 0.030, 95% CI (0.02, 0.44), confirming a positive direct effect of CSEs on leaders’ SelfCare behavior.

**Table 2:** Regression coefficients, standard errors, and model summary information for the conditional process model depicted in Figure 2.

		Consequent						
		Exhaustion				SelfCare behavior		
Antecedent		Coefficient	*SE*	*p*		Coefficient	*SE*	*p*
CSEs	*a*	−0.604	0.063	<0.001	*c*_1_’	0.229	0.104	0.030
Exhaustion		–	–	–	*b*	−0.217	0.098	0.028
OHC		–	–	–	*c*_2_’	0.043	0.088	0.630
CSEs × OHC		–	–	–	*c*_3_’	−0.031	0.103	0.764
Gender^a^	*f*_1_	0.113	0.139	0.420	*g*_1_	0.078	0.168	0.641
Constant	*i*_1_	−0.156	0.197	0.428	*i*_2_	−0.107	0.238	0.652
		*R*^2^ = 0.387				*R*^2^ = 0.175		
		*F*(2,146) = 48.783, *p* < 0.001				*F*(5,143) = 5.090, *p* < 0.001		

*N = 149; one case was deleted due to missing data. Variables were standardized prior to analyses. CSEs, core self-evaluations; OHC, organizational health climate. ^a^Gender is coded as 1 = male, 2 = female.*

However, we did not find a moderating effect of OHC on the relationship between CSEs and SelfCare behavior, *c*_3_’ = −0.031, *p* = 0.764, 95% CI (−0.23, 0.17). Thus, we did not find support for Hypothesis 2.

In line with Hypothesis 3, we found that CSEs were negatively related to exhaustion, *a* = −0.604, *p* < 0.001, 95% CI (−0.73, −0.48). Thus, the more positive leaders’ self-evaluations were, the less exhausted they felt. Similarly, exhaustion was negatively related to SelfCare behavior, *b* = −0.217, *p* = 0.028, 95% CI (−0.41, −0.02), showing that the less exhausted the participants were, the more they engaged in self-directed health behavior (see Table 2 again for both results). A bias-corrected 95% bootstrap confidence interval – based on 10,000 bootstrap samples – for the indirect effect (*ab* = 0.131) excluded zero (0.01, 0.25), thus supporting that CSEs affected SelfCare behavior via exhaustion: Leaders with high CSEs experienced less exhaustion, which was subsequently associated with higher SelfCare behavior.

## Discussion

Leaders’ self-directed health behavior plays a pivotal role in the health of both leaders and their employees. The aim of this study was to investigate the personal antecedents (i.e., CSEs) of leaders’ SelfCare behavior because knowing about antecedents can help make SelfCare behavior more likely to occur. We further wanted to shed light on why, and under which conditions, leaders’ personality affects their health behavior.

Our results showed that CSEs were positively related to SelfCare behavior (direct effect) and that reduced exhaustion mediated this positive relationship (indirect effect), providing support for Hypotheses 1 and 3. Thus, the higher the CSEs of the leaders were, the more SelfCare behavior they engaged in. In addition, when leaders had higher levels of CSEs, they experienced less exhaustion, and this in turn led to more SelfCare behavior. The first result confirmed the idea that leaders’ personality is relevant to their health behavior. More specifically, the way leaders appraise themselves influences the way they take care of themselves. This result is in line with studies that have investigated the associations between the specific traits that are part of CSEs and health-related behaviors. However, this study extends research on personality and health behavior by showing that CSEs as a higher order factor impact leaders’ health behavior.

The finding that reduced exhaustion mediated the relationship between CSEs and SelfCare behavior offers a better understanding on the link between CSEs and health behavior. The CSEs-health link has already been identified multiple times in various contexts (for a review, see Chang et al., 2012), but possible mediational processes had yet to be clarified. The exhaustion-health behavior relationship shown in the present study is in line with the assumption of the CoR theory. Leaders who feel exhausted do not have enough resources to invest time and effort into taking care of their health. Unfortunately, this could start a vicious circle: Engaging in healthy behavior would help leaders to build new resources; avoiding to do so will lead to further loss of resources. Our finding dovetails with research by Byrne et al. (2014), who found that leaders who suffer from depressive symptoms and anxiety lack the resources to care for their employees. Feeling too exhausted to care for oneself or to care for employees is, however, bound to lead to magnify present problems.

We did not find that context acted as a moderating variable. OHC did not make a difference. From a theoretical point of view, this result is surprising. TAT provides a relevant theoretical framework to support the idea that organizational climate moderates the relationship between personality and work behavior. In addition, the approach/avoidance framework provides rational reasons for why OHC should be trait-relevant in terms of leaders’ CSEs. Because of their approach tendency, high-CSE individuals should be more motivated than low-CSE individuals to engage in positive work-related behaviors (i.e., SelfCare behavior) that are aimed at ensuring positive outcomes for themselves. But this might be the point: People with high levels of CSEs act on the basis of their own motivation, which means that they are *intrinsically* motivated to pursue their goals (Chang et al., 2012). Regardless of the quality of the prevailing health climate, they are likely to follow their own agenda and do not need to be activated by situational cues to engage in health behaviors. By contrast, low-CSE individuals generally tend to be motivated by avoidance and tend to remain passive rather than expose and engage themselves.

### Limitations and Future Research

This study has some limitations that need to be mentioned. First, we used a cross-sectional design to test our hypotheses. Because of this, the results need to be interpreted with caution with respect to causality. Thus, reversed effects between CSEs and SelfCare behavior are possible. Leaders’ SelfCare behavior might affect the way leaders evaluate themselves in terms of self-worth, competence, and capabilities. Consequently, future research should apply a longitudinal design to test the proposed model. Besides, an experimental study would add to our understanding of causality in this relation.

Second, we used solely self-report questionnaires for data collection which were all answered by the leaders themselves. Hence, our results rely on a single source and could thus be biased by the presence of common-method variance (Podsakoff et al., 2003). Although the results of Harman’s single-factor test suggest that CMV is not a major concern in this study, method biases may still have an impact. In future research, it would thus be worthwhile to control for CMV by considering “procedural remedies” (Tehseen et al., 2017, p. 146) such as including other data sources, e.g., ratings from colleagues or subordinates. In addition, objective measures, for example, blood pressure data would be helpful as a measure of exhaustion. Finally, predictors and criteria should be measured at two points in time.

Third, the reliability of the SelfCare behavior scale was relatively low. This can be attributed to the fact that the scale measures a broad range of behaviors. The items comprise rather diverse aspects of health behavior (e.g., improving working conditions versus balancing work-life resources) which limits internal consistency but is of course advantageous with regard to validity (i.e., reliability-validity tradeoff). Moreover, the reliability found in this study is comparable to other studies. For example, Franke et al. (2014) found in a sample of employees in Germany a Cronbach’s alpha of 0.67. Future research should nevertheless try to replicate the findings using a health behavior scale with better reliability.

Fourth, we included leaders from a wide range of organizations. Results concerning the moderating effect of OHC may be different when focusing on one organization only. In this case, it would be possible to aggregate individual perceptions of climate to form an index of climate at the unit, team, or organizational levels of analysis (Schneider et al., 2017). Future research should thus collect additional data to take a deeper look into how OHC interacts with leaders’ personality in predicting their SelfCare behavior. Beyond this, future research could benefit from investigating additional moderators of the relationship between CSEs and SelfCare behavior. Whereas OHC refers to the organizational level, possible cues on the social and task-related level are likely; for example, the perceived quality of the relationship between a leader and his or her employees on the team level (leader-member exchange Graen and Uhl-Bien, 1995) or the task complexity a leader is confronted with on the task level.

Fifth, the sample size in this study was quite small. Future research should replicate the findings in a larger leader sample. In addition, a possible self-selection bias restricts the generalizability of our results. It is certainly possible that the leaders who decided to participate in our study consisted primarily of those who were relatively well-equipped to cope with their demands.

### Practical Implications

Our findings offer relevant practical implications for organizations. For example, organizations should invest in personnel development to foster leaders’ CSEs, and thus their health. For example, personnel development measures could be aimed at improving leaders’ self-evaluations in group settings or individual coaching sessions. In addition, organizations could offer SelfCare behavior trainings to support leaders in developing suitable behavioral strategies that will help them take care of themselves. Franke et al. (2011), for example, developed a training concept for leaders that focuses on leaders’ SelfCare as a first step so that leaders will be better able to deal with the their employees’ health in a second one.

Given that OHC was not found to moderate the relationship between CSEs and SelfCare behavior, it does not seem necessary to derive practical implications with respect to this matter. However, it seems rather unlikely that a positive OHC would have negative effects on organizational outcomes. In fact, in our study, there were medium-sized correlations between OHC and the relevant constructs. In addition, other studies have shown that a positive organizational climate might not have only moderating effects but also positive direct effects on different organizational outcomes (Schneider et al., 2017). Thus, organizations should still invest resources into policies, practices, and procedures that are aimed at promoting the health of their members.

## Conclusion

SelfCare behavior can be seen as one way for leaders to stay healthy and fit. Studying leaders’ SelfCare behavior and its personal antecedents is important for leaders and employees alike because it influences the health and well-being of both groups.

In this study, we investigated the direct effect of CSEs on SelfCare behavior in a sample of leaders. We also looked at mediating and moderating processes in order to better understand why and how this relationship occurs. We concentrated on (reduced) exhaustion as a mediator and OHC as a moderator of the CSEs-SelfCare behavior relationship.

Our results showed that CSEs were positively related to leaders’ SelfCare behavior. Leaders with high CSEs engaged more strongly in SelfCare behavior than others, a finding that emphasizes the importance of broad personality traits in health behaviors. Furthermore, we were able to clarify the relationship by showing that reduced exhaustion mediated this relationship. High-CSE individuals experienced less exhaustion, which in turn made SelfCare behavior more likely to occur.

Organizational health climate did not moderate the positive direct effect of CSEs on SelfCare behavior. In future studies, researchers should investigate the role of OHC in the personality-health behavior relationship further and extend studies to capture other possible moderating variables. Future research is needed to replicate and build on our findings with a longitudinal research design.

## Ethics Statement

We have submitted the paper to the ethics committee of the University of Bamberg. This is an online study in which participants took part on a voluntary basis. There is no risk involved for participants.

## Author Contributions

AS and CK planned the study. CK collected and analyzed data, and wrote the first draft of the manuscript. AS discussed the findings with CK and revised the draft.

## Conflict of Interest Statement

The authors declare that the research was conducted in the absence of any commercial or financial relationships that could be construed as a potential conflict of interest.

## Footnotes

1 https://www.uni-bamberg.de/kap/
